# Visual Lateralization in Wild Striped Dolphins (*Stenella coeruleoalba*) in Response to Stimuli with Different Degrees of Familiarity

**DOI:** 10.1371/journal.pone.0030001

**Published:** 2012-01-13

**Authors:** Marcello Siniscalchi, Salvatore Dimatteo, Anna Maria Pepe, Raffaella Sasso, Angelo Quaranta

**Affiliations:** Department of Animal Production, University of Bari “Aldo Moro”, Bari, Italy; Bowling Green State Universtiy, United States of America

## Abstract

**Background:**

Apart from findings on both functional and motor asymmetries in captive aquatic mammals, only few studies have focused on lateralized behaviour of these species in the wild.

**Methodology/Principal Findings:**

In this study we focused on lateralized visual behaviour by presenting wild striped dolphins with objects of different degrees of familiarity (fish, ball, toy). Surveys were conducted in the Gulf of Taranto, the northern Ionian Sea portion delimited by the Italian regions of Calabria, Basilicata and Apulia. After sighting striped dolphins from a research vessel, different stimuli were presented in a random order by a telescopic bar connected to the prow of the boat. The preferential use of the right/left monocular viewing during inspection of the stimuli was analysed.

**Conclusion:**

Results clearly showed a monocular viewing preference with respect to the type of the stimulus employed. Due to the complete decussation of the optical nerves in dolphin brain our results reflected a different specialization of brain hemispheres for visual scanning processes confirming that in this species different stimuli evoked different patterns of eye use. A preferential use of the right eye (left hemisphere) during visual inspection of unfamiliar targets was observed supporting the hypothesis that, in dolphins, the organization of the functional neural structures which reflected cerebral asymmetries for visual object recognition could have been subjected to a deviation from the evolutionary line of most terrestrial vertebrates.

## Introduction

Brain lateralization i.e. the different specialization of the left and right hemisphere is a phenomenon widespread among different animals [1]. Although several studies have reported the presence of both motor and sensory lateralization in aquatic mammals, at present very little information concerning cetecean functional asymmetries in the wild is available [2], [3]. Laterality in visual sensory domain has been reported in many species (fish: [1]–[7] chick: [8]–[11] dog: [12]) and, overall, results supported the general hypothesis that asymmetries in visual perception reflect the different specialization of the right (analysis of novelty/higher emotional valence stimuli) and the left (analysis of familiar stimuli) brain hemispheres. Visual analyses in bottlenose dolphins showed a general superiority of the right visual field (left hemisphere) for visual stimuli discrimination and for visual spatial tasks [13]–[16]. In accordance with these findings, Killian [17] reported a right-visual field advantage for discriminating relational dimensions between stimuli differing in numerosity in a two-choice discrimination paradigm. More recently, the influence of familiarity on the preferential use of one eye to look at human visual stimuli were tested in five captive dolphins and results showed that, at group level, dolphins preferentially use their left eye to look at both familiar and unfamiliar humans [18]. Regarding studies on behavioural laterality in the wild, several studies reported a right-side-down bias during feeding behaviour in gray whales [19] hump-back whales (bottom feeding) [20] and coastal bottlenose dolphins [21]–[23] which could be directly caused by laterality of eye use (right eye→left hemisphere→control of feeding behaviour). Moreover, two studies focused on the visual laterality of social interactions in wild cetacean. The first reported a population-level left-eye use in wild Indo-Pacific bottlenose dolphin (*Tursiops aduncus*) during flipper-to-body rubbing, in which one dolphin (“rubber”) rubs the body of another (“rubbe”) with its flipper [3]; the second report showed a similar left-eye preference during calf-mother interactions in wild belugas whales (*Delphinapterus leucas*), indicating that analysis of socially significant visual information occurs in both dolphins and whales in the right brain hemisphere [2]. Overall, these data demonstrate that asymmetries of eye-use in response to a visual stimulus could be influenced by stimulus characteristics (familiarity, novelty, complexity) [12], [4], [8] as well as subjects' characteristics (age, social environment) [18].

The novel aspect of this study was to investigate visual lateralization in striped dolphins (*Stenella coeruleoalba*) in response to objects of different degrees of familiarity “in the wild”.

## Materials and Methods

### Ethics Statement

The experiments were conducted according to the protocols approved by the Italian Minister for Scientific Research in accordance with EC regulations. No special permission for behavioral research on wild animals such as this study is required in Italy. The committee that allows research without special permission in regard to using animals is the Comitato Etico per la Sperimentazione Animale (University of Bari “Aldo Moro”).

### Study area, observation conditions

The study area, approximately 1.350 km^2^ wide, is situated in the northern portion of the Gulf of Taranto, Ionian Sea portion delimited by the Italian regions of Calabria, Basilicata and Apulia. The bathymetric profile of the Gulf of Taranto is characterised by a central canyon 1000–2000 m deep, and a steep continental slope in near shore waters west of Taranto. Bottom depth within the study area is up to 800 m and primary production seems to be generally higher than in other parts of the Ionian Sea, as a result of significant upwelling [24], [25].

### Survey effort and data collection

Data were collected between April 2008 and September 2011 during weakly surveys from 5.5–6.5 m research vessels equipped with 70–115 HP four-stroke outboard engines. Surveys started from the port of San Vito, south of Taranto, and ended there and were conducted only in good weather conditions. Binoculars were not used to look for cetaceans during navigation, but could be used to confirm species identification whenever necessary. Elevation of observer's eye was about 1.5 m above the sea level.

**Figure 1:**
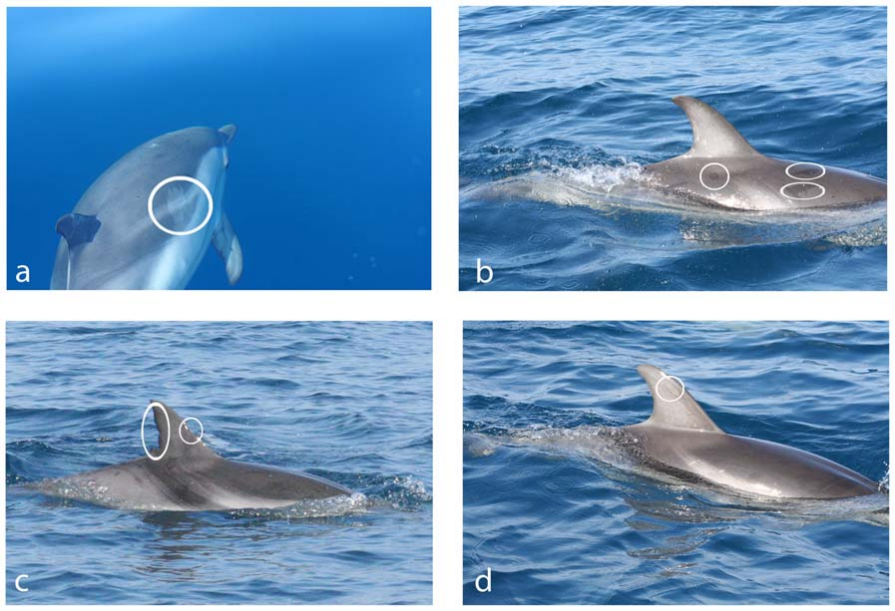
Individual identification. Characteristic markings on the body used for dolphin individual identification (white circles): a-b-d) scratches of different colors; c) fin injuries.

**Figure 2:**
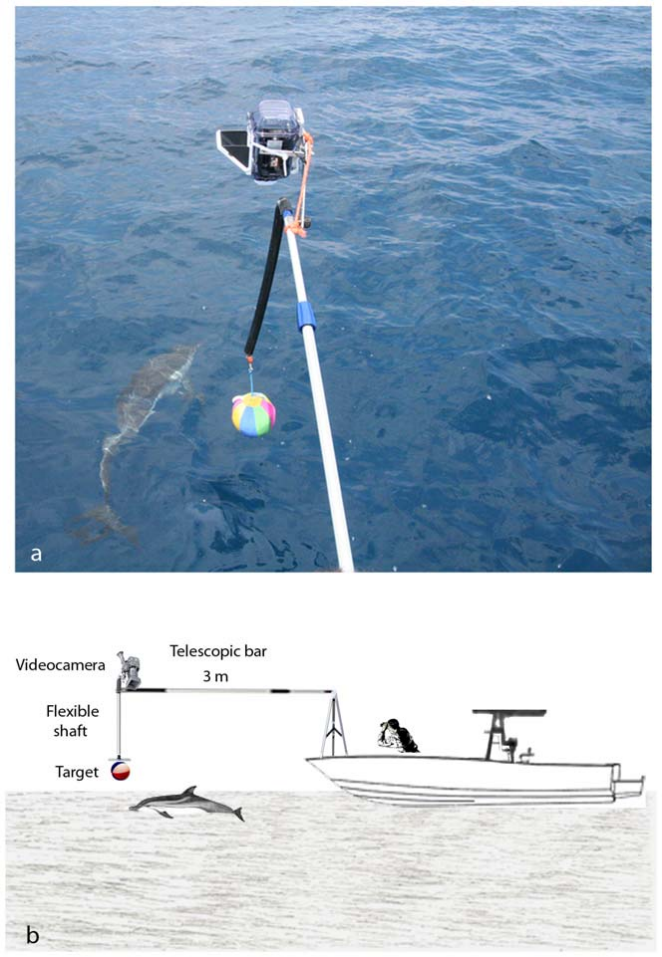
Testing apparatus. Experimental setup: a) Striped Dolphin approaching to the testing apparatus; b) Schematic representation of the testing apparatus, lateral view.

**Figure 3:**
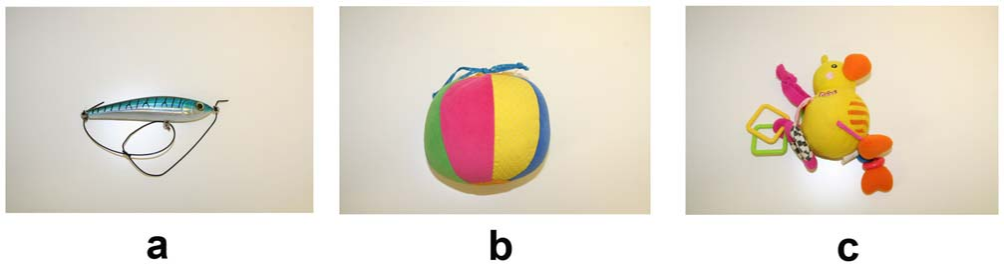
Targets. Visual stimuli: a) blue-fish life-size plastic model; b) coloured ball; c) fabric toy.

**Figure 4:**
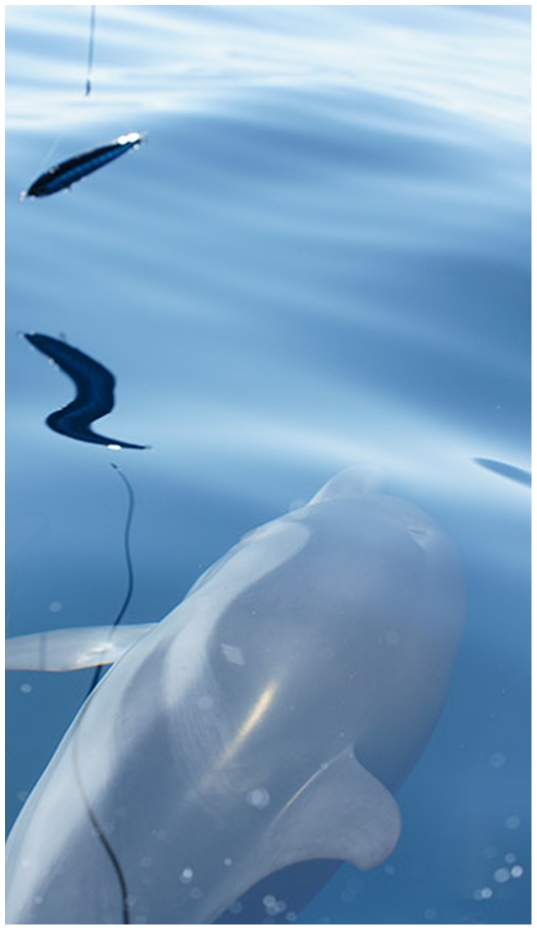
Preferential eye-use during visual inspection of a target. Striped dolphin “#12-Benny” inspecting the fish stimulus using the left eye.

Digital photos and high definition video recordings of the animals taken during the sightings were analysed for detecting natural markers (scars, coloration pattern, fin injuries) necessary for individual identification (Fig. 1). During every single survey the course of the research vessel was set in parallel with dolphin's route and only visual inspection following active engagement with the stimulus (direct approach with the video recording area by the dolphin) were analysed (Fig. 2 a). Different stimuli were presented in a random order by a telescopic bar (length 3.5 m) connected to the prow of the boat (Fig. 2 a–b). The stimuli were a life-size plastic model of a blue-fish, a coloured ball and a fabric toy (Fig. 3). Stimuli were in turns hung up at the tip of the telescopic bar throughout a flexible shaft at a distance of 3 meters from the prow of the research vessels at approximately 10 cm from the sea level (see Fig. 2 b). Dolphins' behaviour was than recorded using a digital video camera superimposed to the tip of the telescopic bar in a way that the recording area was centred on the stimulus (Fig. 2 b). The preferential use of the right/left monocular viewing during inspection of the stimuli was than analysed using a frame by frame technique (Fig. 4).

The sampling method used in this research was an ad lib protocol [3]. No episodes in which two or more dolphins were at same time in the video recording area were analysed.

## Results

During the total experimental period we observed 349 episodes of targets' visual inspections by dolphins.

In these recordings, we observed 244 episodes of visual interactions with targets conducted by 86 identified individuals.

**Figure 5:**
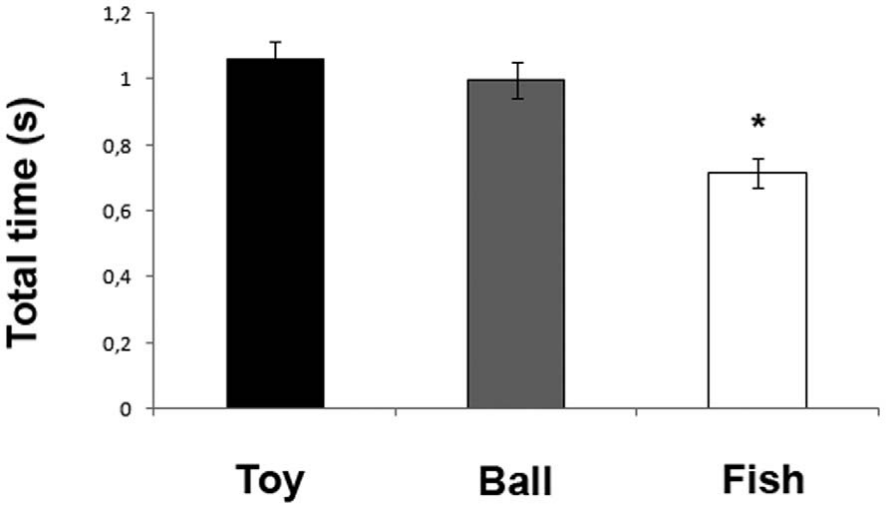
Total time spent looking at different targets. Total time spent during visual inspection of toy (black histogram), ball (gray histogram) and fish (white histogram) stimuli (means with S.E.M. are shown; * P<0.01, Mann-Whitney U-test).

Total time spent looking at different targets was significantly affected by the type of the visual stimulus (H(2) = 24.369, P = 0.000): post-hoc analysis (Mann-Whitney U-test) revealed that this main effect of stimulus was due to the response to the “fish” (mean ± S.D.: 0.71±0.43 s) stimulus being different from the responses to the “ball” (mean ± S.D.: 0.99±0.52 s) and the “toy” (mean ± S.D.: 1.05±0.51 s) stimuli (Fish Vs Toy: U = 2380.00, Z = −4.626, P = 0.000; Fish Vs Ball: U = 2896.00, Z = −3.875, P = 0.000; Toy Vs Ball: U = 3330.50, Z = −0.985, P = 0.324) as can be seen from Figure 5.

**Figure 6:**
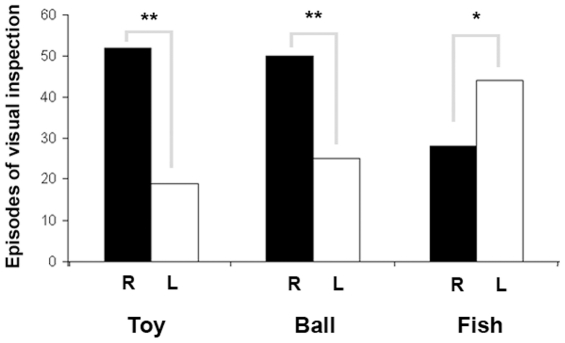
Eye preference to look at different targets. Preferentially right (black histograms) and left (white histograms) eye use during visual inspection of different visual stimuli (means with S.E.M. are shown; * P<0.05; ** P<0.01, Wilcoxon's signed-ranks test).

**Figure 7:**
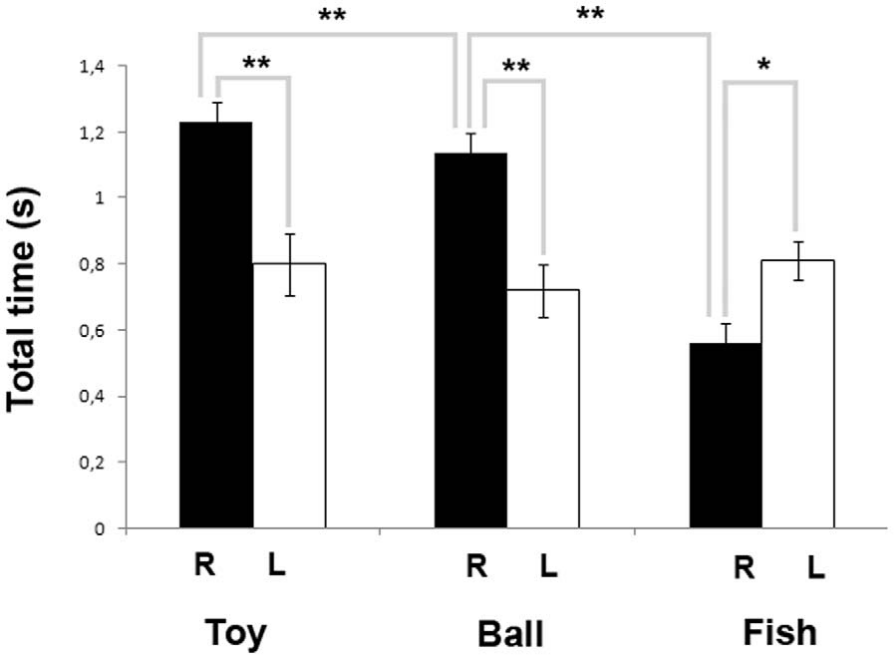
Eye preference to look at different targets - Total time. Total time spent using either the left (white histograms) or right (black histograms) eye during visual inspection of targets (means with S.E.M. are shown; * P<0.01; ** P<0.001, Mann-Whitney U-test).

The bias in the eye-use during visual inspection of different visual stimuli is represented in Figures 6 and 7.

Results revealed a significant effect of the type of the stimulus on the number of right eye-visual interaction with the targets (H(2) = 15.859, P = 0.000): specific between stimuli contrasts (Mann-Whitney U-test) revealed that the number of visual inspection was lower for fish stimulus (mean ± S.D.: 0.78±0.22 s) respect to toy (mean ± S.D.: 1.65±1.13 s) and ball (mean ± S.D.: 1.56±1.12 s) stimuli (Fish Vs Toy: U = 396.000, Z = −3.021, P = 0.003; Fish Vs Ball: U = 415.500, Z = −3.758, P = 0.000; Toy Vs Ball: U = 602.500, Z = −0.068, P = 0.946).

A significant effect of the type of the stimulus was also observed for the number of left eye-visual interactions with different targets (H(2) = 18.536, P = 0.000): post-hoc analysis (Mann-Whitney U-test) revealed that this main effect of stimulus was due to the number of left-eye interactions with fish stimulus being higher (mean ± S.D.: 1.39±0.86 s) respect to toy (mean ± S.D.: 0.57±0.17 s) and ball (mean ± S.D.: 0.78±0.14 s) stimuli (Fish Vs Toy: U = 409.000, Z = −2.899, P = 0.004; Fish Vs Ball: U = 379.000, Z = −4.204, P = 0.000; Toy Vs Ball: U = 546.500, Z = −0795, P = 0.427).

Regarding the right-eye interaction time with different targets, statistical analysis revealed a main effect of stimulus (H(2) = 23.889, P = 0.000): post-hoc analysis (Mann-Whitney U-test) revealed that dolphins spend less time looking with their right eye at fish stimulus (mean ± S.D.: 0.56±0.37 s) respect to toy (mean ± S.D.: 1.23±0.43 s) and ball (mean ± S.D.: 1.14±0.50 s) stimuli (Fish Vs Toy: U = 133.000, Z = −4.547, P = 0.000; Fish Vs Ball: U = 185.500, Z = −4.276 P = 0.000; Toy Vs Ball: U = 1187.500, Z = −1.326, P = 0.185).

No significant effect of the type of the stimulus on the total visual interaction time of left-eye with targets was detected (H(2) = 0.875, P = 0.646).

Regarding the within stimulus analysis, results revealed that dolphins used preferentially their right eye respect to the left during visual inspection of “toy” (toy: right eye = 50 episodes of visual interaction with the target (67%); left eye = 25 episodes of visual interaction with the target (33%), Wilcoxon's signed-ranks test, R = 108.00, N = 32 individuals, Z = −2.410, P = 0.008) and “ball” targets (ball: right eye = 52 episodes of visual interaction with the target (73%); left eye = 19 episodes of visual interaction with the target (27%), Wilcoxon's signed-ranks test, R = 9.00, N = 38 individuals, Z = −4.464, P = 0.000). On the other hand, for “fish” target subjects used their left eye significantly more frequently during visual inspection (fish: right eye = 28 episodes of visual interaction with the target (39%); left eye = 44 episodes of visual interaction with the target (61%), Wilcoxon's signed-ranks test, R = 126.00, N = 41 individuals, Z = 2.659, P = 0.016). Total time spent using either the left or right eye is shown in Figure 7. In line with data reported above on the number of episodes of left/right eye visual interaction with targets, results revealed that dolphins spend significantly more time attending to fabric toy (toy: right eye = 1.23±0.43 s (mean ± S.D.), left eye = 0.80±0.53 (mean ± S.D.); Mann-Whitney U-test, U = 347.00, Z = −4.367, P = 0.000) and ball (ball: right eye = 1.14±0.50 s (mean ± S.D.), left eye = 0.72±0.44 (mean ± S.D.); Mann-Whitney U-test, U = 460.50, Z = −3.780, P = 0.000) targets using their right eye (Fig. 7).

Finally, the duration of visual interaction with the “fish” target was significantly longer when the left eye was used (mean ± S.D.: 0.81±0.45 s), than when the right eye was used (mean ± S.D.: 0.56±0.37 s; Mann-Whitney U-test, U = 755.00, Z = −2.780, P = 0.007) (Fig. 7).

## Discussion

Our results suggest that different stimuli evoked different patterns of eye use in wild striped dolphins: the fish target tended to be viewed with the left eye whereas both the toy and the ball targets were viewed with the right.

Due to the complete crossover of the optic chiasm in dolphins, a right eye bias during visual inspection of “toy” and “ball” targets reflects an overall left hemisphere dominance for visual object processes confirming what has been reported previously in the bottlenose dolphin (*Tursiops truncatus*) and other cetaceans [26]–[30]. In particular, a clear right eye/left hemisphere advantage in a pattern discrimination and acquisition task was reported in adult bottlenose dolphins tested under monocular condition (the animal had to discriminate between simultaneously presented pairs of different patterns with a rubber eyecup fixed onto one of the subjects eyes) [14], [15]. In addition, several studies on dolphins housed in circular tanks reported a strong counterclockwise swimming direction bias: using this particular route, dolphins placed their right eye towards the enclosure wall and thus towards any events outside the pool which could be of importance for the dolphins favouring the activation of the left hemisphere when approaching or scrutinising objects [13]. More recently, Delfour and Marten [16] reported an advantage of the dolphins right visual field when processing different visual stimuli displayed on an underwater touch-screen (two-dimensional figures, three-dimensional figures and dolphin/human video sequences) supporting a left hemispheric dominance in visual information analysis.

In contrast, a right eye/left hemisphere bias occurred during visual inspection of “fish” shaped target. This result is quite interesting since “fish” model represented the target with the highest degree of familiarity for dolphins. In a similar way, Sovrano [4] reported that, when accustomed to the presence of artificial stimuli, fish (*Xenopoecilus sarasinorum*) showed a left bias only when presented with a familiar version of these stimuli, but not when presented with an unfamiliar version.

Overall our findings supported the hypothesis that, in dolphins, asymmetries in eye use during analysis of visual objects reflected a different specialization of brain hemispheres for visual scanning processes which is directly related to the amount of familiarity of the target: the initial learning process of a specific visual pattern (e.g. object's parts and their spatial relationships) have to be encoded separately before creating a stored structural description. This process that is mainly under the control of the left hemisphere (local details of stimuli) seems to occur in our experiment during right eye use in response to toy and ball targets (unfamiliar objects). On the other hand, when object's form has become familiar, its global shape can be directly matched to information stored in memory by configurational analyses (right hemisphere→global stimulus analysis) and this could explain the use of the left eye/right hemisphere in response to the fish target (familiar stimulus) [31]–[33]. Intriguingly, a different complementary specialization of the two hemispheres has been observed repeatedly in the vertebrate brain, in a variety of species (e.g., fish: [4], birds: [8]), with the right hemisphere taking charge of novel information followed by the left hemisphere taking charge of behaviour during visual analysis of familiar stimuli (see for reviews of evidence: [1], [8], [34], [35]). Thus, our data in line with the work of Killian et al [13] supported the hypothesis that, in dolphins, the organization of the functional neural structures which reflected cerebral asymmetries for visual object recognition could have been subjected to a deviation from the evolutionary line of most terrestrial vertebrates.
